# Suicidal ideation associated with depression and social support: a survey-based analysis of older adults in South Korea

**DOI:** 10.1186/s12888-021-03423-8

**Published:** 2021-08-18

**Authors:** Bum Jung KIM, Taesuk KIHL

**Affiliations:** 1 Department of Social Welfare, Chung-Ang University, Seoul, South Korea; 2 Graduate School, Sangmyung University, Seoul, South Korea

**Keywords:** Depression, Social support, Suicidal ideation, Older adults, South Korea

## Abstract

**Background:**

This study examined the effect of depression on suicidal ideation among older adults in South Korea. Furthermore, this study investigated how social support, as a factor that reduces depression among older adults, mediates the relationship between depression and suicidal ideation.

**Methods:**

Based on a survey of 260 older adults in Seoul and Gyeonggi Province, suicidal ideation, depression, and social support were evaluated using the Beck Scale for Suicidal Ideation, the Center of Epidemiological Studies Depression Scale, and the Measurement of Social Support in the Elderly, respectively.

**Results:**

A multiple regression analysis confirmed that depression and social support were significantly associated with suicidal ideation. Based on path analysis, we found that social support mediated the relationship between depression and suicidal ideation.

**Conclusions:**

Therefore, this study provides concrete insights for policymakers and social workers about how suicidal ideation among older adults may be diminished. Particularly, the role of depression and social support in suicidal ideation is a matter of concern for older adults in South Korea.

## Introduction

South Korea (hereafter, Korea) is gradually becoming a super-aged society, with the world’s fastest aging population [1]. In 2017, the older population accounted for 13.8% of the total population, and 6.6% of the older adults lived alone in 2016. In 2026, 20.8% of the population is expected to be aged above 65 years [2]. The impact of this compressed aging phenomenon will be felt at the social and national levels, and inevitably cause various complex social problems. This problem is not unique to Korea but is a global issue in developed countries that must be resolved.

The explosive economic and social changes in Korean society have diminished the status and role of older adults at a faster rate than elsewhere, while there has been a significant increase in the older population. Older adults’ approach to problem solving within the family based on traditional norms has a positive effect on these adults’ daily life. However, the older population is perceived as an unproductive and dependent social group, resulting in older adults being isolated and alienated from other generations [3].

One of the most serious problems in an aging society is suicide among older adults. Korea’s suicide rate among older adults is the highest among the Organization for Economic Cooperation and Development (OECD) member countries. In Korea, the suicide rate of those aged 65 years and above is estimated to be 64.2 per 100,000, which is 2.3 times higher than that of other age groups [4].

In his theory of suicide, Durkheim [5] reported a close relationship between age and suicide, pointing to the negative impact of weakening social integration on the lives of older adults as a possible cause. After retirement, older adults lose their position in the workplace, and their social networks with colleagues are reduced. Following retirement, they often want to spend more time with their spouses and families; thus, integration in and belonging to other social groups gradually weaken. This occurs alongside a gradual loss of economic power and physical ability. Consequently, older adults may encounter negative emotional and psychological conditions, such as alienation, loneliness, isolation, lack of social contact, and lack of community participation due to the collapse or weakening of social integration. In response, some are likely to consider suicide.

Studies have identified depression as an important factor among the causes of suicidal ideation among older adults [6]. Conwell [7] revealed that most older adults who attempted suicide suffered from depression. A 2017 survey of older adults by the Ministry of Health and Welfare reported that 21.1% of all respondents had depression, categorized into the following sub-groups: 65–69 years (15.1%), 70–74 years (18.2%), 75–79 years (23.6%), 80–84 years (30.7%), and above 85 years (33.1%) [8]. The rate of depressive symptoms tended to increase with age. Generally, older adults have a slightly depressive or depressive condition due to physical weakness, loss of role, and regrets over their earlier life [9]. Depression (major depressive disorder) is a condition that requires clinical treatment due to the seriousness of the symptoms. Depression is the most common psychiatric disorder among older adults and is considered treatable. However, if left untreated, it causes chronic complications and results in a very high mortality rate. Depression leads to a decrease in cognitive, physical, social, and emotional functioning and poor quality of life; its manifestations include maladaptation in daily life, headaches, vomiting, physical symptoms (e.g., muscle pain), deterioration of cognition, and problems with social relationships, which may lead a person to attempt to commit suicide. Since depression among older adults can have catastrophic results and even lead to suicide if untreated, it is important to understand the relationship between depression and suicidal ideation. Preventive or regulatory factors should be examined to prevent depression that could cause suicidal ideation or suicide attempts among older adults.

The previous literature indicates that depression greatly influences suicidal ideation; however, not every older adult exposed to such situations transitions to suicidal ideation and actions. Factors such as social support, social participation, social integration, and self-integration are effective in preventing depression from developing into severe depression and in buffering the effects of depression [10–12]. Studies on the relationship between social support and suicidal ideation have shown that social support is a major factor in reducing suicidal ideation [13, 14] and impacts the process of forming suicidal ideation [15, 16]. In addition, social support has been reported to play a mediating and regulatory role in reducing the negative effects of depression or stress that trigger suicidal ideation [17, 18].

This study examined the effect of older adults’ depression on suicidal ideation, which has been suggested as a strong influencing factor on this demographic’s suicidal ideation. Furthermore, this study investigated how social support, as a factor that reduces depression among older adults, mediates the relationship between depression and suicidal ideation.

## Literature review

### Depression and suicidal ideation

O’Connell et al. [19] described the process leading to suicide in five steps: “passive desire to die” → “suicidal thoughts” → “suicide plan” → “suicide attempt” → “suicide.” Suicidal thoughts are thoughts related to the intentional termination of one’s life that have not yet been carried out [9]. Some claim that suicidal thoughts do not directly lead to suicidal behavior [20]; however, other studies show that frequent suicidal thoughts are likely to increase the likelihood of suicide [21, 22]. Suicidal ideation does not necessarily lead to suicide, but it is a powerful predictor of suicidal action and suicide completion. Therefore, understanding the wealth and justice factors related to suicidal ideation among older adults could help prevent suicide by minimizing situations in which suicidal behavior occurs. Factors related to suicidal ideation among older adults include depression [23, 24], life stressors [25], social support [26], economic factors [27], psychological factors [28, 29], disease and physical factors [30, 31], and drug or alcohol abuse [32].

Depression is the most proximal correlate of suicidal ideation and suicidal behavior among older adults, regardless of the presence of other factors; the higher the depression, the higher the suicidal ideation [6, 24]. According to the 2011 National Mental Illness Epidemiology Survey [33], the lifetime prevalence of major depressive disorder in the general population in South Korea is 6.7%. A total of 24.9% of the respondents had experienced a major depressive disorder, including serious suicidal ideation, and 35.8% were experiencing a major depressive disorder when they attempted suicide [33].

Osgood [34] stated that depression among older adults is characterized by helplessness and hopelessness. Older adults are prone to despair, since they cannot control their own life events and neither they nor anyone else can do anything to help them change their situation of helplessness and pain. Various factors, such as demographic, biological, psychological, economic, and social, are involved in complex ways in older adults’ depression. Regarding demographic factors, the following have been shown to increase depression among older adults: higher age, being male, lack of a spouse, unemployment, and a low economic and educational level [35, 36]. In addition, psychological factors, such as decreased self-esteem due to reduced status and role in the family and society, are considered significant causes of depression among older adults [28, 37]. Moreover, social isolation is related to depression, and a vicious circle of social isolation is exacerbated by symptoms of depression [38].

Jang and Kim [39] found that stress among older adults affects depression, which acts as a major factor in propelling suicidal ideation. Waern et al. [40] showed that the impact of depression on suicide attempts and suicidal ideation was stronger in older than in younger adults. Kim [41] argued that suicide among older adults is caused by multiple factors rather than a single factor and, similar to other studies, stated that depression is a greater risk factor for suicide among older adults than any other age group. In Kim’s [41] research on the correlation between variables of social integration, exchange resources, depression, and suicide, he found that depression directly affects suicide, while the social integration variables of family and community integration and the exchange resource variables of health and economic status affect it indirectly. He confirmed that the mediation affects suicide but depression has a direct effect on suicide variables.

### Social support as a mediator between depression and suicidal ideation

Social support is related to self-esteem, and is directly associated with depression among older adults. When social support is shown to have a significant impact on depression or psychological well-being [42], or when the integration into the family and community to which the older adult belonged is weakened, older adults fall into isolation or experience depression [43]. High social support reportedly reduces depression, and thereby, suicide [44].

Social support comprises all the positive resources individuals can obtain from their interpersonal relationships. The structural domain of social support refers to the network of connections in an individual’s social environment [45]. Social networks with various characteristics are formed according to the content, quality, and quantity of relationships [46]. In addition, social support promotes the expression of positive emotions and trust from others through the social network. It generates positive actions, such as receiving recognition from others, obtaining information and advice to solve problems, and accessing economic and material assistance. Kahn and Antonucci [47] described the function of social support in terms of emotional, informational, material, and evaluation support. Cohen and Wills [48] divided social support into two aspects: emotional support that provides encouragement and sympathy to others and instrumental support that helps to solve problems and accomplish tasks. In functional terms, social support consists of tools, information, and emotions [49]. Instrumental support refers to practical assistance, such as providing necessary material assistance. Informational support refers to providing necessary information, such as giving advice or listening. Emotional support includes intimacy, concern, and consideration. In addition, self-awareness and self-esteem can be strengthened through emotional support.

The effects of social support can be either direct or moderating. In a study on the effect of social support on stress, the direct effect of social support produced a positive effect regardless of the level of stress factors [50]. Furthermore, when a stressful situation arises, an individual’s positive emotions, stability in life, and positive perception of self-worth have a positive effect regardless of the degree of stress, which constitutes the effect of social support [51]. By contrast, the moderating effect of social support is that it strengthens people’s ability to overcome problems or enhances their behavior when they experience stress [52]. Cohen and Wills [48] explained that the moderating effect of social support occurs at two points in time: when evaluating a particular event and when solving a problem.

A study on the effects of social support on suicidal ideation with depression as a parameter showed that social support related to suicide or suicidal ideation had an indirect effect by influencing depression [44, 53, 54]. Lee [53] found that social support was negatively correlated with depression among older adults and had an indirect effect through depression on suicide among older adults. Similarly, Lee and Cho [54] analyzed the effect of social support on older adults living alone, and stated that although social support has no direct effect on suicidal ideation, it indirectly affects suicidal ideation by influencing depression.

In addition, studies have been conducted on the moderating effect of social support in the relationship between depression and suicidal ideation among older adults [13, 15, 55]. Park [55] showed that suicidal ideation moderately decreases when the family’s level of communication is high; however, depression increases when the level of communication is low and suicidal thoughts rise rapidly. According to Lee [13], family support, as a particular form of social support, has a negative relationship with suicidal ideation among older adults, and social support from the family reduces the relationship between depression and suicidal ideation. Therefore, the present study examined the correlation between depression, suicidal ideation, and social support among older adults as well as the mediating effects of strong social support on depression and suicidal ideation.

## Materials and methods

### Data

Data for the present study were collected from 260 older adults from Seoul and Gyeonggi Provinces in South Korea. Using convenience sampling, the survey was administered at various organizations, including community-based long-term care facilities, such as senior welfare centers, senior community centers, and nursing homes. Totally, 15–20 people were recruited from each of the 10 institutions as participants. Healthcare professionals, such as social workers, helped to distribute the survey questionnaires. The research team first contacted the program directors at social service agencies to explain the main purpose and the complete research procedure to obtain their consent to participate. Later, eligible participants identified through the screening process were recruited to participate in face-to-face interviews and complete the structured questionnaires. The Institutional Review Board of the XXX University (No. xxxx) approved this study. Participation in the study was voluntary. The study adhered to a rigorous protocol for research ethics, guaranteeing participants’ anonymity and confidentiality, and clearly stated that the collected data would only be used for research purposes.

Of the 295 individuals recruited, 260 completed in-depth face-to-face interviews, giving an acceptance rate of 87%. Some individuals dropped out of the study, citing disagreement with its subject or other reasons. There were no missing data, as the in-depth interviews were conducted by trained social workers. Each interview lasted an average of 35 min, and compensation of $5 was provided to each participant upon completion.

### Measures

#### Suicidal ideation (SSI)

Suicidal ideation was measured by the 19-item Scale for Suicidal Ideation (SSI), originally developed by Beck et al. [56]. There are five screening items in the original SSI. Three items focus on the desire to live or die, and two items assess the desire to die by committing suicide. An additional 14 items assess suicidal risk factors, such as the frequency and duration of suicidal thoughts, control over attempts, number of deterrents, and the actual amount of preparation for anticipated attempts. The 19-item SSI has a high internal consistency, with Cronbach’s alpha ranging from .84 [57] to .89 [56]. Each item offers three options that are graded according to suicidal ideation, with scores ranging from 0 to 2, where higher scores indicate higher suicidal ideation [58]. This study used the Korean version of the SSI, translated and used by Park and Shin [59]. Cronbach’s alpha for the SSI administered in this study was .94.

#### Depression (CES-D)

This study used the 20-item Center of Epidemiological Studies Depression (CES-D) scale [60]. The CES-D scale includes questions related to psychological, physical, and emotional symptoms that manifest when people are emotionally distressed. Symptoms include feeling hopeless about the future, feeling helpless, loss of appetite, sleeplessness, and feeling lonely, sad, or blue [61]. Responses range from rarely or none (0) to most of the time (3). Higher scores indicate greater depression. The internal consistency of the scale in the present study was .94.

#### Social support (MOSS-E)

This study used the Measurement of Social Support in the Elderly (MOSS-E), which combines various types of social support, including emotional support, instrumental support, and providing support to others. Emotional support engages compassion and bolsters mental health. Instrumental support affords practical help with activities of daily living, such as cooking, eating, and cleaning. Providing support to others is a crucial component of healthy social relationships. The MOSS-E does not consider the role of the subtypes of social support separately because it was based on a single integrated model. Study participants were scored 1 for *yes* and 0 for *no* in response to 10 questions (e.g., Is there anyone you can easily ask to help you with chores?). A score of 10 indicated that the participant had strong social support [62]. Previous research has tested and validated the MOSS-E scale in assessing social support for both older Asian populations [62–65] and the older Korean population [66, 67]. The internal consistency of MOSS-E in this study (Cronbach’s α) was .86.

#### Sociodemographic variables

Concerning basic attributes, this study examined sociodemographic variables, including age (years), gender (Female = 0, male = 1), marital status (single = 0, married = 1), income, and education (years).

### Statistical analysis

Descriptive statistics were used to determine the basic characteristics of the study sample. In addition, Pearson’s correlations were used to examine the bivariate relationship between independent (depression, social support) and dependent (suicidal ideation) variables to identify strengths and multicollinearity. To analyze the relative effects of independent variables on suicidal ideation, multiple regression was performed with three models (1: sociodemographic, 2: including depression, 3: including social support). Path analysis was employed to examine the mediating effect of social support on the relationship between depression and suicidal ideation. Data were analyzed using Stata version 13.

## Results

### Descriptive characteristics of the sample

Table 1 shows the descriptive characteristics of the sample. Participants’ average age was 75.11 years (SD = 7.85), ranging from 65 to 92 years. Of the 260 participants, 173 were female (66.5%) and 123 were married (47%). Participants’ average monthly income was $1338.8 (SD = 1058.97), ranging from $0–4500. Their average length of education was 10.7 years. Their depression scores ranged from 0 to 36 with an average of 12.6 (SD = 8.73). The average scores on social support and suicidal ideation were 4.10 (range: 0–7.36) and 7.7 (range: 0–40), respectively.

**Table 1:** Descriptive characteristics of the study sample (*N* = 260)

Variable	*N*	%
Age (in years)		
Range	65–92	
Mean (*SD*)	75.11 (7.85)	
Gender		
Female	173	66.54
Male	87	33.46
Marital Status		
Not married	137	52.69
Married	123	47.31
Income (monthly in USD)		
Range	0–4500	
Mean (*SD*)	1338.87 (1058.97)	
Education (in years)		
Range	0–20	
Mean (*SD*)	10.74 (5.02)	
Social Support		
Range	0–7.63	
Mean (*SD*)	4.10 (6.13)	
Depression		
Range	0–36	
Mean (*SD*)	12.61 (8.73)	
Suicidal Ideation		
Range	0–40	
Mean (*SD*)	7.70 (10.27)	

Notes: Gender: Female = 0, Male = 1; Marital Status: Single = 0, Married = 1

### Correlational analysis

Using Pearson’s correlation analysis (Table 2), this study confirmed that multicollinearity was not an issue (variance inflation factors < 3.01). Participants who had experienced higher suicidal ideation were more likely to be older (*r* = .14, *p* < .05), single (*r* = −.19, *p* < .01), and have lower income (*r* = −.20, *p* < .01), lower education (*r* = −.14, *p* < .01), higher levels of depression (*r* = .55, *p* < .01), and lower levels of social support (*r* = .25, *p* < .01).

**Table 2:** Correlations (*r)* among study variables (*N* = 260)

	1	2	3	4	5	6	7
1. Age	–						
2. Gender	.00						
3. Marital Status	−.22**	.53**					
4. Income	−.67**	.06	.27**				
5. Education	−.29**	.29**	.17**	.38**			
6. Social Support	.06	.17**	.11	.02	.23**		
7. Depression	.50**	−.22**	−.37**	−.47**	−.32**	−.14*	
8. Suicidal Ideation	.14*	.02	−.19**	−.20**	−.14*	.25**	.55**

*p < .05, **p < .01; Gender: Female = 0, Male = 1; Marital Status: Single = 0, Married = 1

### Regression model for suicidal ideation

Using multiple regression analysis, three regression models were constructed (Table 3). Model 1 includes the sociodemographic variables that were entered in the first step. The first model explained 10% of the total variance. Among the variables, the association between suicidal ideation and (a) gender (β = .21, *p* < .01), (b) marital status (β = −.26, *p* < .01), (c) income (β = −.12, *p* < .05), and (d) education (β = −.12, *p* < .05) were found to be statistically significant. Participants who were male, single, and had low income and education levels were more likely to experience suicidal ideation. Model 2 (including depression scores) explained an additional 35% of the variance (β = .67, *p* < .00). Finally, Model 3 (including social support scores) explained an additional 6% of the variance, showing that the correlation between social support and suicidal ideation was statistically significant (β = .38, *p* < .05). Participants with higher levels of depression and lower levels of social support were more likely to experience suicidal ideation.

**Table 3:** Multiple regression models of suicidal ideation among older Koreans

Variables	Model 1		Model 2		Model 3	
	Β	*T*-value (***p***)	β	*T*-value (***p***)	β	*T*-value (***p***)
Age	−.04	−.78	−.06	−6.15**	−.11	−5.93**
Gender	.21	2.95**	.12	4.02**	.15	4.23**
Marital Status	−.26	−3.93**	−.12	−1.57	−.10	−1.44
Income	−.12	−2.15*	−.10	−.07	−.08	−.12
Education	−.12	−2.45*	−.01	−.54	−.01	.21
Depression			.67	11.35**	.55	12.24**
Social Support					.38	−2.52*
*R*^2^	.10		.45		.51	
Adjusted *R*^2^	.08		.43		.49	
*F*	7.87**		29.34**		29.16**	

*p < .05, **p < .01

### Path analysis

Although regression analysis showed that the influence of major independent variables (depression, social support) on suicidal ideation was statistically significant, the problem of limited measurability arises, whereby only the direct effects of independent variables can be identified. Thus, path analysis was used to test a comprehensive analytical model for suicidal ideation that included both direct and indirect effects among the key study variables. Table 4 shows the standardized and unstandardized regression weights of each path. Path analysis allowed us to examine both direct and indirect effects. As shown in Table 5, depression was positively related to suicidal ideation. Furthermore, the positive correlation between depression and social support was found to be statistically significant, and social support showed a direct positive association with suicidal ideation. Indirect effects involve chains of straight arrows called compounded paths, where the path along the arrow is always forward [68]. In our study, depression was negatively associated with social support, which in turn led to decreased suicidal ideation.

**Table 4:** Path analysis results

	Unstandardized estimates (SE)	Standardized estimates	*T*-value (***p***)
Social support ← depression	.00	−.01	−2.28*
Suicidal ideation ← social support	.47	−1.68	−3.54**
Suicidal ideation ← depression	.05	.75	13.67**

*p < .05, **p < .01

**Table 5:** Standardized direct, indirect, and total effects predicting suicidal ideation

	Direct effects	Indirect effects	Total effects
Social support ← depression	−.01*		−.01*
Suicidal ideation ← social support	−1.68**		−1.68**
Suicidal ideation ← depression	.74**	.03*	.77**

*p < .05, **p < .01

## Discussion

This study examined the association of depression and social support with suicidal ideation among older adults in South Korea. Given that suicide among older adults in South Korea is a serious social threat and the country has the highest suicide rate for older adults among the OECD countries, the present study’s findings help broaden our understanding of the risk factors related to suicidal ideation. Furthermore, they provide practical and policy implications for developing preventive measures. In summary, depression and social support were found to be statistically significant risk factors for suicidal ideation among older adults in South Korea. In addition, the relationship between age, gender, and suicidal thoughts was found to be statistically significant.

The present study found statistically significant correlation between depression and suicidal ideation. In other words, older Koreans with severe depression are more likely to experience high suicidal ideation levels. This finding is consistent with the previous literature [23, 32]. Regarding preventive methods for reducing depression, screening to assess the risk of depression by professionals, such as physicians, nurses, and social workers, working closely with older clients in community settings can be beneficial. In particular, social workers who work with older clients in various social service agencies play a key role in identifying any emotional changes that may occur among them (e.g., depression level). In addition, social workers who have contact with family members of older clients can detect emotional or relational changes in the family dynamics. In such cases, they can play an important role in screening and enabling the family to have their older relative assessed for depression. Furthermore, mandatory medical check-ups that include depression assessments should be implemented by the government through active links with mental health service agencies and suicide prevention service centers.

Furthermore, this study found that social support was strongly associated with suicidal ideation. According to the previous literature, social support has an indirect effect on suicidal ideation through depression [44, 53, 54]. However, previous studies did not show any significant results on the direct effect of social support on suicidal ideation [54]. Conversely, social support showed a direct positive correlation with suicidal ideation in the present study.

Older Koreans with strong social support are less likely to experience suicidal ideation. Korea’s family structure has changed from an extended to a nuclear family. In addition, the proportion of older adults living alone and that of households solely comprising older couples are rapidly increasing, while the proportion of children supporting their parents and living with them is significantly decreasing. In addition to changes in family structure, various social activities related to work decrease in old age. Moreover, physical constraints on activity increases, weakening the link to former colleagues and other social groups. As this situation continues, the social activity sphere of older adults shrinks and social isolation deepens, resulting in reduced social support. When the social support provided by family members or colleagues is weakened, emotional sympathy with neighbors decreases, making it difficult for older adults to achieve emotional exchanges and seek physical help. Consequently, loneliness and mental stress increase, leading to negative thoughts about life, which in turn can lead to suicidal ideation [61, 69].

While depression among older adults is characterized by feelings of helplessness, i.e., one cannot control their daily life, and hopelessness, i.e., no one can help [34], social support can help alleviate the feeling of hopelessness among this population. Social support, including emotional support, instrumental support, and providing support to others, may play a role in controlling depression, and thereby, reducing suicidal ideation.

It is noteworthy that the multiple regression analysis showed that male, single, low-income, and low-educated participants were more likely to experience suicidal ideation. Concerning the effect of sociodemographic characteristics on depression among older Koreans, previous studies reported that depression was higher among older men and older adults without a spouse and who were unemployed and whose economic and educational statuses were low [35, 36]. The characteristics of the older adults who are highly depressed and those who are more likely to experience suicidal ideation overlap. Therefore, healthcare professionals must actively work to improve the level of social support for this population.

To expand social support, policymakers, healthcare professionals, and social workers must recognize its importance in reducing suicidal ideation. In particular, workers at social welfare institutions should be aware of the important role they play in working closely with the family members and friends of older clients to minimize their emotional vulnerability. Therefore, interventions that promote social contact, support, and integration with families and communities can be effective. For example, telephone support is associated with a marked reduction in suicide among older adults [43]. Family therapy and community educational programs also help to improve family relationships and communication skills. More importantly, social workers at social service agencies should actively provide opportunities for older adults to expand their participation in social activities. They should help to expand the social networks of older adults by providing information about jobs available for seniors and various leisure, education, and volunteer programs in the community. The resulting expansion of their social domain would help to expand older adults’ human networks, which would increase the degree of social support in their surroundings.

## Conclusions

This study found that depression is associated with suicidal ideation among older adults through the mediating effect of social support, which implies that managing social support levels is especially important to prevent suicidal ideation among older adults with depression. Older adults with weak social support should increase their social support levels from both formal and informal caregivers. Healthcare experts should recognize the importance of social support management, with a particular focus on those suffering from depression as a high-risk group for suicide. Finally, it is important for healthcare workers and policymakers to initiate social support extension programs for older adults and screen them for depressive symptoms in medical or mental health settings.

This study has some limitations. First, it is difficult to generalize the findings to the entire older population in Korea because of the relatively small sample size. Using a nationally representative sample to investigate risk factors related to suicidal ideation among older Koreans is recommended in future studies. Second, causal relationships between study variables could not be identified due to the cross-sectional nature of the study. More rigorous future studies applying longitudinal analysis to identify risk factors related to suicidal ideation are required. Third, a limited number of variables were included as risk factors; however, there might be other contributing variables (physical health, functional status, cognitive status). Therefore, a comprehensive study should be undertaken to examine more extensive risk factors in the future.

Despite these limitations, this study provides clear and practical evidence of the links among key risk factors of suicidal ideation, particularly focusing on depression and social support. Furthermore, it provides suggestions for preventive strategies to promote the development of suicide prevention services and social support development programs.

## Data Availability

The datasets used and analyzed during the current study are not publicly available due to some personal data contained within them but may be available from the corresponding author on reasonable request.

## Abbreviations

OECD: Organization for Economic Cooperation and Development
SSI: Scale for Suicidal Ideation
CES-D: Center of Epidemiological Studies Depression
MOSS-E: Measurement of Social Support in the Elderly

## References

[1] Lee HS. Counseling for the elderly. Seoul: Hakjisa; 2012.

[2] National Statistical Office. Future population estimation, 2017. Daejeon: Korea National Statistical Office; 2016. http://kosis.kr/conts/nsportalStats/nsportalStats_0102Body.jsp?menuId=10&NUM=1014. Accessed July, 2020.

[3] Chang KS (2001). Compressed modernity and Reconception of the aging issue: aged people as a new generation. Family Culture.

[4] National Statistical Office. Elderly statistics, 2014. Daejeon: Korea National Statistical Office; 2014.

[5] Durkheim E (1951). Suicide: a study in sociology (Spaulding JA, Simpson G, trans.).

[6] Bonnewyn A, Shah A, Demyttenaere K (2009). Suicidality and suicide in older people. Rev Clin Gerontol.

[7] Conwell Y (1997). Management of suicidal behavior in the elderly. Psychiatr Clin N Am.

[8] Ministry of Health and Welfare. Survey on older adults in South Korea, 2017. Seoul: Ministry of Health and Welfare; 2017. http://www.mohw.go.kr/upload/viewer/skin/doc.html?fn=1533627270504_20180807163432.pdf&rs=/upload/viewer/result/202007/. Accessed July, 2020.

[9] Bae JY, Kim WH, Yoon KA (2005). Depression, suicidal thoughts, and the buffering effect of social support among the elderly. J Korean Gerontol Soc..

[10] Kim MH, Lee GY, Chung S (2000). A path analysis on depression among the elderly. J Korea Gerontol Soc.

[11] Greenglass E, Fiksenbaum L, Eaton J (2006). The relationship between coping, social support, functional disability, and depression in the elderly. Anxiety Stress Coping.

[12] Jang IY, Lee HY, Lee E (2019). Geriatric fact sheet in Korea 2018 from national statistics. Ann Geriatr Med Res.

[13] Lee IJ (2011). Moderating effects of life problems, social support on the relationship between depression and suicidal ideation of older people. Health Soc Welf Rev.

[14] Lee HK, Kwon JH (2015). The influence of grief level on suicidal ideation among bereaved single-household elderly-focusing on moderating effect of social support. Korean J Gerontol Soc Welf.

[15] Vanderhorst RK, McLaren S (2005). Social relationships as predictors of depression and suicidal ideation in older adults. Aging Ment Health.

[16] Bae JH (2011). The buffering effect of psycho-social resources on suicidal ideation of the elderly. J Soc Res.

[17] Hobbs M, McLaren S (2009). The interrelations of agency, depression, and suicidal ideation among older adults. Suicide Life Threat Behav.

[18] Kang JH, Shin TS (2015). The effects of adolescents’ stress on suicidal ideation: focusing on the moderating and mediating effects of depression and social support. Korean J Youth Stud.

[19] O’Connell H, Chin AV, Cunningham C, Lawlor BA (2004). Recent developments: suicide in older people. Br Med J.

[20] Simons RL, Murphy PI (1985). Sex differences in the causes of adolescent suicide ideation. J Youth Adolesc.

[21] Bonner RL, Rich AR (1987). Toward a predictive model of suicidal ideation and behavior: some preliminary data in college students. Suicide Life Threat Behav..

[22] Kumar G, Steer R (1995). Psychosocial correlates of suicidal ideation in adolescent psychiatric inpatients. Suicide Life Threat Behav..

[23] Conwell Y, Caine ED, Olsen K (1990). Suicide and cancer in late life. Psychiatr Serv.

[24] Corna LM, Cairney J, Streiner DL (2010). Suicide ideation in older adults: relationship to mental health problems and service use. Gerontologist..

[25] Kang SH, Moon ES, Cha MY (2011). The structural relationships among life event stress, social support, depression, and suicidal ideation: a comparison of boy and girl high school students. Korean J Educ Psychol.

[26] Schroepfer TA (2008). Social relationships and their role in the consideration to hasten death. Gerontologist..

[27] Kim HS, Kwon LK (2013). Relationship between elderly suicide rates and socio-economic factors in Korea: centering around the trend of changes in 1990–2010. J Korea Contents Assoc.

[28] Shin HG (2012). Elderly’s path model of passing four major pains on to suicidal thought mediated by hopelessness and depression. Korean J Gerontol Soc Welf..

[29] Bae JH, Um KW (2009). Factors affecting suicide attempt of the elderly. J Korean Gerontol Soc.

[30] Harwood DMJ, Hawton K, Hope T, Harriss L, Jacoby R (2006). Life problems and physical illness as risk factors for suicide in older people: a descriptive and case-control study. Psych Med.

[31] Stice BD, Canetto SS (2008). Older adult suicide: perceptions of precipitants and protective factors. Clin Gerontol.

[32] Suominen K, Isometsä E, Suokas J, Haukka J, Achte K, Lönnqvist J (2004). Completed suicide after a suicide attempt: a 37-year follow-up study. Am J Psych.

[33] Ministry of Health and Welfare (2012). 2011 national mental illness epidemiology survey.

[34] Osgood N, Palmore E (1984). Suicide. Handbook on aging in the United States.

[35] Hur JS, Yoo SH (2002). Determinants of depression among elderly persons. Ment Health Soc Work.

[36] Choi SJ, Joo EY, Lee YJ, Hong SB (2015). Suicidal ideation and insomnia symptoms in subjects with obstructive sleep apnea syndrome. Sleep Med.

[37] Kwon JD, Kim DK, Kim KS, Park SJ (2012). Structural relationship of family cohesion, stress, depression, and problem drinking for the elderly. J Soc Sci.

[38] Craft LL, Landers DM (1998). The effect of exercise on clinical depression and depression resulting from mental illness: a meta-analysis. J Sport Exerc Psychol.

[39] Jang MH, Kim YH (2005). The relationship of stress, depression, and suicidal ideation in the elderly. J Korean Acad Psychiatr Ment Health Nurs.

[40] Waern M, Rubenowitz E, Wilhelmson K (2003). Predictors of suicide in the old elderly. Gerontology..

[41] Kim HS (2002). A study on epistemology of Korean elders’ suicidal thought. J Korean Gerontol Soc..

[42] Kwon YS, Oh SJ, Lee SJ (2020). The effects of social support on depression of the elderly: focusing on the moderating effect of leisure activity satisfaction. Korean J Educ Gerontol.

[43] De Leo D, Buono MD, Dwyer J (2002). Suicide among the elderly: the long-term impact of a telephone support and assessment intervention in northern Italy. Br J Psychiatry.

[44] Brown SL, Nesse RM, Vinokur AD, Smith DM (2003). Providing social support may be more beneficial than receiving it: results from a prospective study of mortality. Psychol Sci.

[45] Cohen SE, Syme SL (1985). Social support and health.

[46] Schaefer C, Coyne JC, Lazarus RS (1981). The health-related functions of social support. J Behav Med.

[47] Kahn RL, Antonucci TC, Kiesler SB, Morgan IN, Oppenheimer VK (1981). Convoys of social support: a life-course approach. Aging: social change.

[48] Cohen S, Wills TA (1985). Stress, social support, and the buffering hypothesis. Psychol Bull.

[49] Helgeson VS (1993). Two important distinctions in social support: kind of support and perceived versus received. J Appl Soc Psychol.

[50] Thoits PA (1984). Explaining distributions of psychological vulnerability: lack of social support in the face of life stress. Soc Forces.

[51] Dunkel-Schetter C, Sarason BR, Sarason IG, Pierce GR (1990). Differentiating the cognitive and behavioral aspects of social support. Social support: an interactional view.

[52] Murrell SA, Norris FH, Chipley QT (1992). Functional versus structural social support, desirable events, and positive affect in older adults. Psychol Aging.

[53] Lee MS (2005). Study on the effect of social support on depression and suicide of the old. Korean J Clin Soc Work.

[54] Lee GY, Cho E (2013). A study on the effect of main variables to the suicidal ideation among the elderly living alone: focused on the direct and indirect effects of social support. Health Soc Welf Rev..

[55] Park BG (2008). Study on a moderating effect of psychosocial characteristics in the relationship between depression and suicidal ideation among community elderly. J Korean Gerontol Soc..

[56] Beck AT, Kovacs M, Weissman A (1979). Assessment of suicidal intention: the scale for suicide ideation. J Consult Clin Psychol.

[57] Beck AT, Brown GK, Steer RA (1997). Psychometric characteristics of the scale for suicide ideation with psychiatric outpatients. Behav Res Ther.

[58] Kim BJ, Ahn JH (2014). Factors that influence suicidal ideation among elderly Korean immigrants: focus on diatheses and stressors. Aging Ment Health.

[59] Park KB, Shin MS (1990). College goal and suicidal ideation among high school students. Korean J Clin Psychol.

[60] Eysenck HJ, Eysenck SBG (1975). Eysenck personality questionnaire.

[61] Ahn JH, Kim BJ (2015). The relationships between functional limitation, depression, suicidal ideation, and coping in older Korean immigrants. J Immigr Minor Health.

[62] Harada S, Shu-Chuan T, Sakihara S, Takakura M (2001). The relationship between social support with depressive symptoms and life satisfaction among the elderly in rural community. Ryukyu Med J.

[63] Sakihara S, Harada S, Sakihara S (2000). Revision and prediction validity of the measurement of social support-elderly (MOSS-E). Longitudinal study on the social environment and a long life in Okinawa.

[64] Shima S, Shikano T, Kitamura T, Asai M (1985). New self-rating scales for depression. Clin Psychiatr.

[65] Takizawa T, Kondo T, Sakihara S, Ariizumi M, Watanabe N, Oyama H (2006). Stress buffering effects of social support on depressive symptoms in middle age: reciprocity and community mental health. Psychiatr Clin Neurosci.

[66] Wong ST, Yoo GJ, Stewart AL (2005). Examining the types of social support and the actual sources of support in older Chinese and Korean immigrants. Int J Aging Human Dev.

[67] Wong ST, Yoo GJ, Stewart AL (2007). An empirical evaluation of social support and psychological well-being in older Chinese and Korean immigrants. Ethn Health.

[68] Klem L, Grimm LG, Yarnold PR (1995). Path analysis. Reading and understanding multivariable statistics.

[69] Seo IK, Cho HC (2013). Mediation effects of depression in the relationship between stress and suicidal ideation of the elderly: a comparative study on people who live alone and those who live with family. J Welf Aged.

